# Targeting Innate Immunity to Enhance the Efficacy of Radiation Therapy

**DOI:** 10.3389/fimmu.2018.03077

**Published:** 2019-01-14

**Authors:** Tahir B. Dar, Regina M. Henson, Stephen L. Shiao

**Affiliations:** ^1^Department of Radiation Oncology, Cedars-Sinai Medical Center, Los Angeles, CA, United States; ^2^Department of Biomedical Sciences, Cedars-Sinai Medical Center, Los Angeles, CA, United States

**Keywords:** radiation therapy, innate and adaptive immune response, immunotherapy, macrophages, dendritic cells, NK cells

## Abstract

Radiation continues to play a major role in the treatment of almost every cancer type. Traditional radiation studies focused on its ability to damage DNA, but recent evidence has demonstrated that a key mechanism driving the efficacy of radiation *in vivo* is the immune response triggered in irradiated tissue. Innate immune cells including macrophages, dendritic cells, and natural killer cells are key mediators of the radiation-induced immune response. They regulate the sensing of radiation-mediated damage and subsequent radiation-induced inflammation. Given the importance of innate immune cells as determinants of the post-radiation anti-tumor immune response, much research has been devoted to identify ways to both enhance the innate immune response and prevent their ability to suppress ongoing immune responses. In this review, we will discuss how the innate immune system shapes anti-tumor immunity following radiation and highlight key strategies directed at the innate immune response to enhance the efficacy of radiation.

## Introduction

Radiation (RT) continues to play a major role in the treatment of cancer with more than 50% of all cancer patients receiving RT sometime during their treatment course (1). Traditionally, the primary mechanism of action for RT's effect on tumors was thought to be RT-induced DNA damage to malignant cells. However, recent evidence demonstrating the critical role of the immune system in regulating the response to cytotoxic therapies such as RT has challenged this long-standing assumption about how RT mediates its anti-tumor activity.

Early work from Stone et al. demonstrated that mice lacking T and B cells required more RT to control the same size tumor compared to immune intact animals (2). Other groups have since gone on to show the importance of IFN-γ producing cytotoxic CD8+ T cells (3, 4) as critical effectors in the tumor response to RT. Thus, it has become clear that a T cell response is required for RT to attain its maximal efficacy. However, T cell responses are the culmination of a multi-step inflammatory response that begins with RT-mediated damage to a tumor and its microenvironment. The sensing of this damage and transmission of the signals to generate a productive immune response is the responsibility of the most ancient form of immunity, the innate immune system. The innate immune system that includes natural killer (NK) cells, macrophages and dendritic cells (DCs) serves as the early warning system of body and the gatekeeper to T cell responses. By virtue of its early role in inflammation, innate immunity has the ability to shape the magnitude and character of the RT-induced immune response (summarized in Table 1). We review here how the innate immune system regulates the response to RT and highlight potential therapeutic approaches that target innate immunity in combination with RT to enhance the RT-mediated anti-tumor immune response.

**Table 1:** Summary of immune cells in the tumor microenvironment.

**Innate immune cells**	**Interaction with radiation therapy**	**References**
Dendritic cells 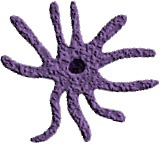	• Batf3-dependent DCs induced by RT promote anti-tumor immune responses by activating CD8 + T cells • DCs upregulate MHC-I after RT, promoting efficient tumor antigen presentation for better anti-tumor immune responses • RT enhances tumor antigen presenting capacity of infiltrating DC through type I IFN production	(5, 6) (6–8) (9)
Macrophages 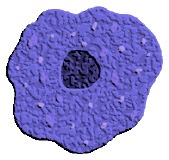	• RT induces TGF-ß and IL-4 leading to production of alternatively activated/M2 macrophages which inhibit anti-tumor immune responses • Macrophages promote matrix remodeling, vasculogenesis which support tumor regrowth post-RT • Inhibiting macrophages via CSF-1R, Axl, Cd11b results in better anti-tumor responses post-RT	(4, 10–13) (14, 15) (4, 16–18)
NK cells 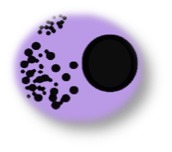	• RT enhances cytotoxic activity of NK cells against various solid tumors including pancreatic cancer and sarcoma	(19–21)

*Innate and adaptive immunity play many roles in the context of tumor biology. Key functions of each of the immune cells is listed*.

### Innate Immunity

The immune system is often separated into two categories: innate and adaptive immunity. The key distinction between the categories is antigen specificity, i.e., the ability of each cell to uniquely recognize and respond to a single specific molecular entity. Adaptive immunity consisting primarily of B and T cells provide the diverse specificity of the immune system through the essentially infinitely rearrangeable B and T cell receptors. Innate immunity largely composed of dendritic cells, myeloid/macrophages and natural killer (NK) cells provide the *context* for an immune response through a specialized set of receptors designed to distinguish when a given target poses a danger and should be eliminated by the immune system (22). Upon recognition of a common array of molecular patterns called pathogen associated molecular patterns (PAMPs) or danger-associated molecular patterns (DAMPs) which signal the presence of pathogens or tissue-damage (“danger”), the innate immune system initiates an immune response (23). Cells of the innate immune system serve not only as early responders to contain the source of inflammation, but also as the gateway to a full and robust immune response by transmitting critical signals to activate the adaptive immune system. Once the combination of the earlier innate immune response and the later adaptive response have eliminated or contained the source of antigen, the innate immune system, particularly the myeloid cells/macrophages, helps restore tissue homeostasis by clearing dead cells, restoring the vasculature and reconstituting the normal tissue structure (24). Thus, given the innate immune system's critical role in the initiation, maintenance, and resolution of an immune response, it is no surprise that the innate immune system plays an important role in regulating the immunobiology of tumors affecting everything from the progression of tumors to their response to therapy.

## Radiation Therapy and Innate Immunity

**Figure 1:**
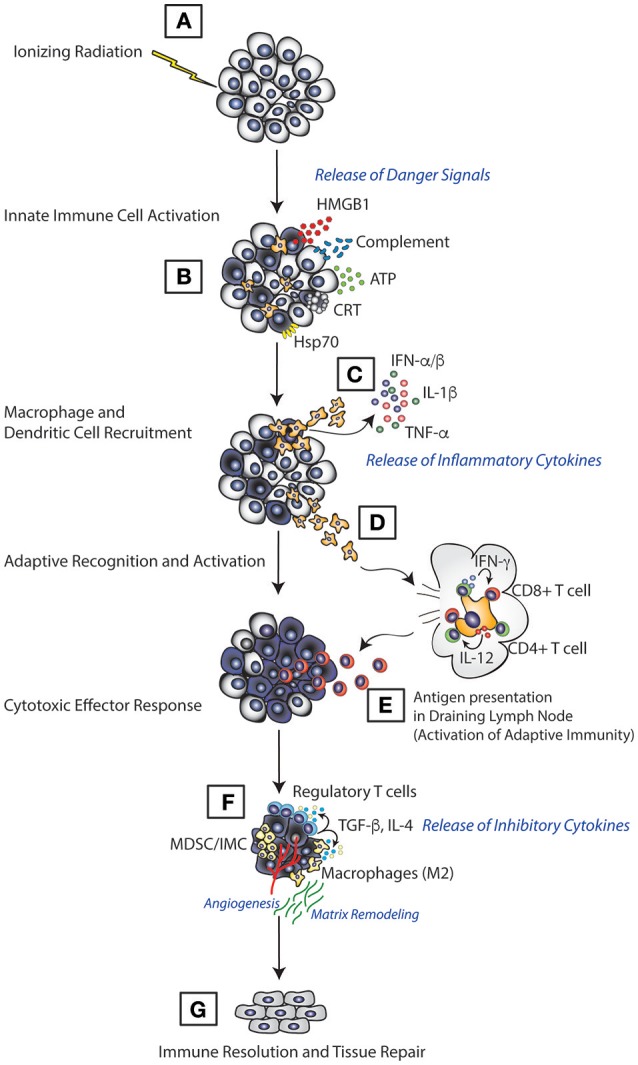
Model of immune activation following RT. RT induces direct tumor cell death which leads to release of various immunological mediators in the form of ATP, HMGB1, calreticulin, and complement **(A)**. This leads to innate immune cell priming, where innate immune cells, such as dendritic cells and macrophages, recognize these mediators through various receptors, migrate to the tumor **(B)** and induce the production of inflammatory molecules such as TNF-α, IL-1β, and type I IFN **(C)**. Innate cells then migrate to the lymphoid tissue **(D)** carrying antigens acquired from the tumor cell for presentation **(E)** resulting in activation of the adaptive immune response and elimination of tumors. Once tumors are eradicated, the RT-induced inflammation is suppressed **(F)**, and tissue damage associated with tumors and the immune response is repaired **(G)**.

Among cancer therapies, RT possesses unique biology as a result of its ubiquity in the environment. Given the omnipresent nature of radiation from natural sources such as naturally occurring isotopes and cosmic radiation, all organisms from bacteria to humans have had to develop methods to deal with cells damaged by irradiation. Activation of the innate immune system is one of those methods and likely serves as one of the main mechanisms driving the extraordinary efficacy of RT. Evidence of the importance of innate immunity in the response to RT come from studies that demonstrate reduced efficacy for RT in preclinical models of cancer which are deficient in innate immune cells including NK cells (25), macrophages (4, 16), and DCs (26). These findings are further supported by numerous observations from patients; one study in hepatocellular carcinoma, for example, showed that increased numbers of circulating myeloid cells following RT correlated with poorer responses (27). Thus, given that innate immunity has such an important role in determining the response to RT, multiple groups have explored the mechanisms by which RT interacts with the innate immune system. We discuss the findings from these studies below in the context of the different functions of the innate immune system: initiation of inflammation, activation of the adaptive immune response and resolution of an immune response (Figure 1).

### Role of Radiation in the Initiation of an Anti-tumor Immune Response

**Figure 2:**
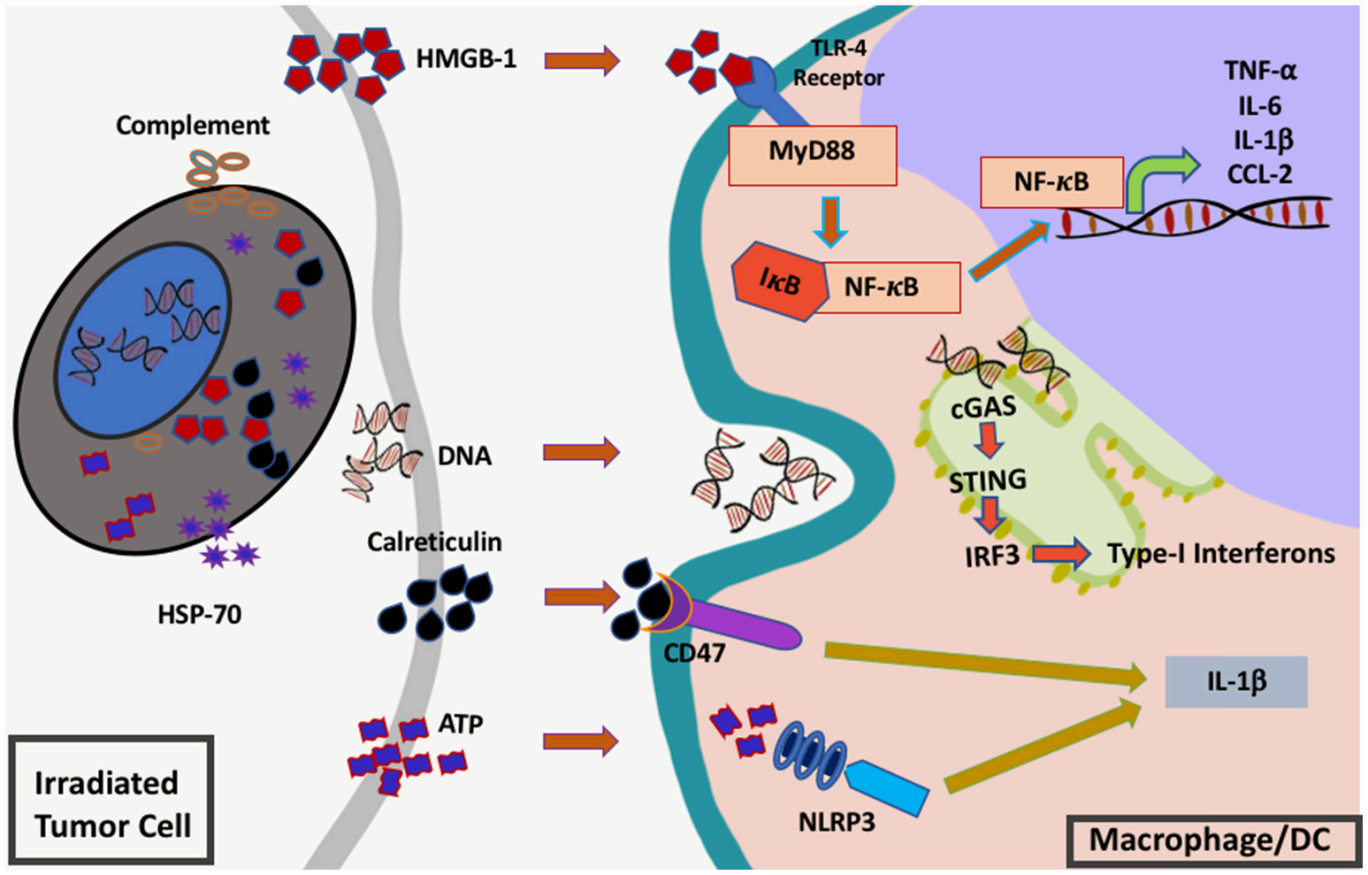
Innate immune signals (“danger signals”) triggered by RT. RT induces the release and activation of multiple different inflammatory mediators from injured cells including complement, heat shock protein 70 (hsp70), high-mobility group box protein 1 (HMGB1), cytosolic DNA, calreticulin, and adenosine triphosphate (ATP). These molecules are sensed by innate immune cells such as macrophages or dendritic cells via toll-like receptor 4 (TLR-4), cyclic GMP-AMP synthase (cGAS)-stimulator of interferon genes (STING), CD47 and NLR family pyrin domain containing protein 3 (NLRP3). Once sensed these receptors send signals via nuclear factor kappa B (NF-κB) and interferon regulatory factor 3 (IRF3) leading to downstream cytokine production and subsequent inflammation.

As previously mentioned, one of the primary functions of the innate immune system is to regulate the initiation of an immune response. In the sterile environment of most tumors, innate immune cells initiate an immune response following detection of signals that indicate the presence of cell damage or danger. Radiation activates the innate immune system by inducing both tumor and normal cells to release specific danger signals that leads to activation of multiple inflammatory pathways in innate immune cells. These danger signals include high-mobility group B-1 (HMGB1), calreticulin, complement, and cytosolic DNA all of which act upon receptors on innate immune cells and lead to release of mediators such as cytokine and chemokines that trigger an immune response (28–30) (Figure 2).

The HMGB1 protein is a nuclear protein that is released by damaged cells and binds to toll-like receptor 4 (TLR4), the main receptor for lipopolysaccharide (LPS). Thus, HMGB1, like its bacterial counterpart LPS, can stimulate macrophages and dendritic cells which express high levels of TLR4 leading to cytokine production and upregulation of molecules (MHC, B7.1, B7.2) that lead to activation of T cells. It was one of the first inflammatory molecules identified in the setting of RT. Apetoh et al. demonstrated that RT releases HMGB1 and that depletion of HMGB1 or loss of TLR4 reduced the efficacy of RT (29). Interestingly, they also identified a variant in the TLR4 gene that leads to less efficient binding and patients with the variant seemed to do worse with standard of care therapy which in many instances included a course of RT (28). In addition to HMGB1, calreticulin (CRC) has also been shown to be expressed on the surface of cells following RT leading to better anti-tumor immunity (30). Calreticulin serves as a phagocytic signal for macrophages which engulf the dying cells and subsequently can present tumor antigens (31). TLR4 is highly expressed on innate immune cells, thus the primary responders to RT-associated HMGB1 are likely macrophages and DCs in the tumor microenvironment (28, 30). Further, macrophages as the primary cells responsible for the clearance of damaged cells are responsible for recognizing calreticulin. Thus, for the extracellular inflammatory signals produced by RT, the innate immune system serves as the main conduit to conduct danger signals to the rest of the immune system.

Recent studies have also identified cytosolic DNA as a critical inflammatory signal induced by RT (32, 33). Of the various cancer therapies, RT, in particular, damages DNA both directly and indirectly within the nucleus and mitochondria and in doing so generates DNA fragments both with the nucleus and cytosol. Cytosolic DNA is recognized by an intracellular protein called cGAS (cyclic GAMP synthase) which leads to production of cGAMP (2′-5′ GMP-AMP). cGAMP activates the endoplasmic reticulum (ER)-bound STING (stimulator of interferon genes) pathway which further recruits and phosphorylates TBK1 (TANK-binding kinase 1), leading to phosphorylation and activation of IRF3 (IFN-regulatory factor 3) and subsequent production of Type I interferons like IFN-β (Figure 1) (34, 35). cGAS and STING are highly expressed by a variety of innate immune cells such as macrophages, dendritic cells, and others and required for optimal production of type I interferons (36–38). Recent evidence from several groups have shown that the cGAS-STING pathway is responsible for detecting cytosolic tumor–derived DNA after RT-induced damage to the DNA (32, 34, 39, 40). Subsequent production of type I interferons post-RT are critical for generating the anti-tumor cytotoxic CD8+ T cell response. Studies in murine B16 melanoma model revealed that the surge of IFNβ production in irradiated tumors is associated with enhanced RT-induced anti-tumor effects in IFN receptor intact mice which is lost in mice lacking the IFN receptor (IFNAR-1^−/−^) (9, 41). Other DNA damaging agents such as anthracyclines have also been shown to signal through the cGAS-STING-IFN pathway to produce anti-tumor immune responses (42).

Recent observations have shown that a DNA exonuclease called 3′ repair exonuclease 1 (Trex1) regulates RT-induced activation of the cGAS-STING-IFN pathway. Using paired RT-sensitive and resistant orthotopic breast cancers it was revealed that RT-sensitivity depends in part on Trex1 levels. Mechanistically, Trex1 cleaves the DNA that accumulates in the cytosol following RT thereby abrogating IFN-β production through the STING-cGAS pathway. Thus, high levels of Trex1 prevent radiation-induced Type I interferon induced inflammation thereby reducing the efficacy of RT (5). Interestingly, multiple smaller fractions of radiation (8 Gy^*^3) did not induce higher levels of Trex1, rather it induced more IFN-β production and activation of Batf3-dependent DCs, leading to enhanced anti-tumor T cells responses. The induction of Trex1 by a single fraction of high-dose radiation dose but not with a short-course of fractionated radiation suggests that it may be essential to fractionate the radiation doses to improve the immunogenicity of RT and its synergy with immunotherapy. Preclinical studies and a recently reported clinical trial support this notion demonstrating synergy between fractionated RT and anti-CTLA (43). In the checkpoint-resistant breast TSA model (mouse), it was observed that single high dose (20 or 30 Gy) of RT did not induce abscopal effects when used along with either anti-CTLA-4 or anti-PD-1 while a short fractionated course (8 Gy^*^3) induced an abscopal systemic immune response when given in conjunction with anti-CTLA-4 leading to prolonged/sustained tumor regression. Fractionated lower doses (8 Gy^*^3) induced the production of IFN-I stimulated genes in mice followed by enhanced number of CD8α+ tumor infiltrating DCs (with high CD70) within the tumors and IFNβ in TSA cells *in vitro* but interestingly 20 Gy did not in part through in the induction of Trex1 by high-dose single fraction RT. Trex1 knockdown in TSA cells restored Type I interferon production with high doses of radiation (20 Gy^*^2) suggesting that induction of Trex1 is a key mediator of RT-induced inflammation. Thus, to ensure an optimal anti-tumor responses, short-course fractionated RT may need to be employed in part to prevent Trex1 induction leading to optimal sensing of the cytosolic DNA produced by RT.

By sensing an array of danger signals produced by irradiated cells, innate immune cells serve as the primary sentinels of the body to identify cells that have been damaged by radiation (Figure 1). As such, the innate immune system plays an outsized role in determining the response to radiation damage. Macrophages and dendritic cells integrate the danger signals they received from irradiated cells and their response to these signals shapes the ensuing immune response. Thus, many strategies are currently being explored to augment the response of the innate immune system following RT to help create better anti-tumor immunity.

### Targeting Innate Immune Initiation of an Anti-tumor Immune Response

Most of the strategies directed at augmenting the innate immune response have focused on increasing signals that mimic the danger signal sensed by the innate immune system. In preclinical models, these strategies have shown much promise and are beginning to be tested clinically. The oldest and most common strategy that has been utilized to enhance the early innate immune response typically targets toll signaling. While RT naturally leads to release of HMGB1 which binds TLR4, other toll agonists have also been utilized to augment the inflammatory response triggered by RT. For example, addition of CpG, a TLR9 agonist, showed synergy when combined with RT (44) in a murine and canine models of melanoma and a murine model of breast cancer (45). Imiquimod, a TLR7 agonist, also showed increased activity in conjunction with RT in several different murine models of cancer (46, 47) with enhanced immune activation noted in a trial of human breast cancer skin metastases (48). In one preclinical study, topical application of imiquimod to lesions in a murine breast cancer model in combination with RT and low-dose cyclophosphamide led to tumor regression for both the irradiated and distant lesions and was further associated with an upregulation of IFN-α and IFN-γ signaling and CD8+ T cell homing to the tumor site (49). Similarly, systemic administration of another TLR7 agonist, DSR-6434 resulted in enhanced radiation efficacy with prolonged tumor regression in murine models of colorectal carcinoma and fibrosarcoma with increased type I interferon production (47).

Other strategies to improve innate immunity have focused on the calreticulin pathway. As mentioned previously, calreticulin expression is induced by RT and is an important signal for phagocytosis by macrophages (31). This process is regulated in part by a molecule known as CD47 (integrin associated protein) which interacts with signal regulatory protein-alpha (SIRPα) expressed on myeloid cells. This interaction causes phosphorylation of the SIRPα cytoplasmic immunoreceptor tyrosine-based inhibition motifs and recruitment of Src homology 2 domain-containing tyrosine phosphatases to ultimately result in delivering an anti-phagocytic signal to myeloid cells preventing a cell from being consumed (50). While, not acting directly in concert, the phagocytic stimulation provided by RT-induced calreticulin can be enhanced by blocking the anti-phagocytic signal CD47 which leads to increased dendritic cell and macrophage activation and improved anti-tumor immunity (51, 52). Trials are currently underway testing this pathway in combination with RT (ClinicalTrials.gov, NCT02890368).

One of the most promising newer strategies to augment the RT-mediated activation of innate immunity has been to target type I interferon production through the use of STING agonists. Mostly structured as cyclic dinucleotides, multiple groups have shown the efficacy of STING agonists in combination with chemotherapy and various immunotherapies (53–55). STING agonists in combination with RT have also been examined (56, 57) and in these murine models of pancreatic cancer and prostate cancer, STING agonists in combination with RT showed significant synergy. In this study using a murine model of pancreatic cancer, Baird et al. found that RT along with STING agonist-CDN displayed strong synergy significantly enhancing tumor regression through augmented CD8+ T cell responses (57). Similar synergy was also observed in murine models of lung cancer (LLC) and colorectal cancer (MC38) (56). Like the other agents targeting cancer by augmenting innate immune activation, STING agonists are currently in early phase clinical trials for multiple different cancer types (ClinicalTrials.gov, NCT03172936).

While limited clinical information exists, there is substantial preclinical data suggesting that augmenting the innate immune activation triggered by RT can significantly enhance the anti-tumor immunity produced following RT (Figure 1). However, given the significant release of these innate immune activating molecules following RT at baseline there may be other aspects of how innate immune cells interact with tumors that can serve as additional targets.

### RT and Regulation of the Anti-tumor Adaptive Immune Response

When cells of the innate immune system detect that there is a problem, e.g., an infection or tissue damage, they activate a program of inflammation that leads to activation of the adaptive response (T and B cells). Activation of the adaptive immune system requires maturation of dendritic cells or macrophages into antigen-presenting cells (APC) which requires appropriate expression of MHC molecules and co-stimulatory signals. Interestingly, RT has been shown to upregulate MHC class I and stimulate presentation of unique antigens (7, 8, 58) as well as costimulatory molecules (58, 59) by dendritic cells. The importance of DC in mediating the efficacy of RT was shown Dewan et al., where fractionated radiotherapy along with anti-CTLA4 had significant abscopal effects in part through the generation of increased numbers of Batf3 DCs (43). Batf3 dependent DC cells are an important subset of dendritic cells with their ability to efficiently cross-present antigens and regulate tumor growth by enhancing CD8+ T cell migration to the tumor microenvironment and fostering effective T cell response (6, 60). Abscopal effects were abolished in the Batf3^−/−^ mice consistent with other observations demonstrating the critical role of Batf3 DC in regulating RT-induced anti-tumor immune responses (60–62). In addition to its effects on DC, RT further contributes to the adaptive immune response by encouraging innate immune cells to establish an inflammatory milieu in irradiated tissue in part through stimulating the release of complement and pro-inflammatory cytokines and chemokines by innate immune cells (63, 64).

Following innate recognition, one of the first proinflammatory molecules activated by RT is complement, soluble effector proteins that are produced by and regulate innate immune cell function (65, 66). Surace et al. demonstrated that components of the complement system are important for RT-induced anti-tumor immunity both in murine and human tumors (66). They showed higher levels of activated C3a and C5a (inflammatory anaphylatoxins) in tumors within 24 h of RT and that these mediate the response to RT-induced damage to tumor cells (66). They went on to demonstrate that in a mouse melanoma model, DC activation post-RT was dependent on these anaphylatoxins. In their model, DC activation post-RT was only observed in wild-type mice but not in mice lacking C3, the C3a receptor or the C5a receptor. Previous studies have shown that anaphylatoxins can bind on their own receptors (67, 68), thus, following RT it was observed that DC increased the expression of some of the complement factors including C3 and the C5a receptor within 24 h following RT and that expression of these complement factors were critical for controlling DC activation and subsequent T-cell responses following RT. As would be expected from a complement response (69), RT-mediated complement activation increased NK1.1+ (natural killer cells) but not NKp46+ (invariant NK-T cell) cell populations which likely served to enhance anti-tumor response of CD8+ T cells.

In addition to expression of complement, RT has been shown to increase the expression of a number of cytokines and chemokines. Aside from the previously mentioned type I interferons, RT has been shown to induce immune cells within the tumor including TAMs and CD8+ T cells and NK cells and others to produce inflammatory cytokines including tumor necrosis factor alpha (TNF-α) (70), interleukin-1 (71), interleukin-6 (72, 73), interferon-gamma (IFN-γ) (3, 74), macrophage colony stimulating factor 1 (CSF-1, M-CSF) (75), and granulocyte macrophage colony stimulating factor (GM-CSF) (76, 77). These cytokines are critical for establishing inflammation at the irradiated site as well as induction of a cytotoxic CD8+ T cell response. Genetic ablation or use of agents that deplete or block the actions of theses cytokines significantly reduced the response to RT across a number of histologies including melanoma, sarcoma and breast in murine models. These cytokines not only serve to attract circulating immune cells, but also help establish inflammation by altering the vasculature (78) and increasing the release of chemokines including CXCL16 (79, 80), CCL2 (81), and CCL5 (82). These chemokines serve to attract CD8+ T cells (CXCL16) and myeloid cells (CSF-1, CCL2, CCL5) to irradiated tumors.

Through the expression of various inflammatory molecules, the innate immune system translates the danger signals they sense in irradiated tissue into an anti-tumor immune response. Multiple strategies have been employed combining RT with various agents in attempt to enhance the innate immune response to RT as we discuss below.

### Enhancing Innate Regulation of the Anti-tumor Adaptive Immune Response

In addition to targeting the danger signaling induced by immunogenic cell death, multiple groups have sought to make the downstream responses of the innate immune cells more productive. Strategies to enhance the magnitude and efficiency of antigen presentation and inflammation induced by RT are currently being explored.

The primary target of the strategies to augment the innate immune response following RT has been focused on dendritic cells as they are the primary APC within tumors. Several groups have shown that they can improve the response to RT in murine models and early human trials by increasing the growth and differentiation of dendritic cells. One way to increase the number of DC is the cytokine granulocyte-macrophage colony-stimulating factor (GM-CSF) which has been shown to be a crucial pathway for the growth, maturation and migration of DC (83, 84). Several human trials of GM-CSF in melanoma and breast cancer have demonstrated the efficacy of GM-CSF administration alone with improved survival compared to historical controls (85) (86) and an increase in circulating DC (87). Based on these successful early studies, trials of GM-CSF and RT were initiated. In one trial of metastatic patients of various histologies, exogenous administration of GM-CSF with a course of fractionated RT (35 Gy in 10 fractions) found evidence of an abscopal, and hence systemic, anti-tumor immune response in 27% of the patients (84). Currently, multiple trials are underway to test the efficacy both locally and systemically of combining GM-CSF with high-dose, short-course radiation (stereotactic body radiation therapy, SBRT) in hepatocellular carcinoma (ClinicalTrials.gov, NCT02946138) and lung cancer (ClinicalTrials.gov, NCT02976740, NCT02623595, NCT03113851) and standard fractionated RT in glioblastoma (ClinicalTrials.gov, NCT02663440).

Another cytokine for DC-specific growth similar to GM-CSF that has been shown to enhance the response to RT is the FS-like tyrosine kinase 3 ligand (FLT3L) (88–90). FLT3L binds and activates FLT3 on hematopoetic progenitors and serves a critical role in steady-state maintenance of DC (91) and increased levels of FLT3L during inflammation mobilizes DC (92). Two studies using preclinical models of non-small cell lung cancer demonstrated reduced tumor growth, metastases, and improved survival with administration of RT and FLT3L in a T-cell dependent manner (89, 90). Based on the success of the preclinical data, FLT3L is currently being tested in a phase II trial in non-small cell lung cancer in combination with SBRT (ClinicalTrials.gov, NCT02839265). Preclinical data in a murine model of hepatocellular carcinoma has also shown that the efficacy of RT can be enhanced by augmenting DC function through the use of exogenous IL-12 to help DCs better generate cytotoxic T cells (93).

Instead of encouraging the creation of more or better DCs, others have taken a more direct approach and have tested combining dendritic cell vaccines with RT (94, 95). In two trials for glioblastoma, DC loaded with tumor lysates were administered either concurrently with chemoradiation (96) or immediately following (97) demonstrated increased numbers of tumor specific T cells, however neither showed correlation between immune response and survival, though they were not powered enough to determine such a correlation. Currently, an open trial in brainstem glioma combines both of the above strategies employing both DC vaccination, GM-CSF, and standard RT for patients (ClinicalTrials.gov, NCT03396575). Though previous trials have not been able to show significant survival impact using the combination of DC targeting with RT, as understanding of the underlying immune mechanisms increases more combinations with various immune therapies as well as different doses and timing of RT may further enhance the response to DC vaccines and RT.

While none of these strategies have had tremendous clinical responses to date, the advent of newer immunotherapy approaches particularly those targeting tumor immunosuppression such as checkpoint inhibitors have generated renewed interest in RT and DC vaccination combinations.

### RT and Innate Immunity-Mediated Immunosuppression

The recent success of agents known as checkpoint inhibitors that block immunosuppressive pathways within tumors highlight the importance of targeting the immunosuppressive tumor microenvironment to foster anti-tumor immunity. Cells of the innate immune system particularly macrophages in conjunction with tumor cells participate in establishing the suppressive environment of tumors. Macrophages play a complex dual role in the context of tumor immunobiology. They have pro-inflammatory roles as outlined above, but more often exhibit a pro-tumor phenotype that suppresses anti-tumor immunity and supports tumor growth (98, 99). In the context of radiation therapy, multiple groups have shown that macrophages play a negative role in regulating the anti-tumor response after RT thus reducing the efficacy of RT. Several groups have reported increased numbers of myeloid-macrophages migration following RT in models of head and neck cancer, glioma, pancreatic, and breast cancer (4, 16, 81, 100, 101). Further, many of these macrophages have been shown to have an immunosuppressive pro-tumor phenotype, also known as the M2 or alternatively activated phenotype, which limits the response to RT (4, 102, 103). Further, macrophages are one of the key cells within tumors that express both PD-1 (104) and PD-L1 (105). Thus, given the role of innate immune cells like macrophages as sources of tumor immunosuppression, it is not surprising that many groups have explored targeting the suppressive capacity of innate immune cells to improve the efficacy of RT.

### Targeting Innate Immunity-Mediated Immunosuppression in Combination With RT

With the recent recognition of the need to alleviate the intrinsic tumor immunosuppression to allow anti-tumor immunity to progress, much activity has been devoted to targeting the pathways and cells that mediate immunosuppression. Interestingly, many of the cellular targets are innate immune cells such as macrophages. Since RT generates both an anti-tumor immune response and the corresponding suppressive immune control mechanisms, combinations of RT with agents that target intratumoral immune suppression are thought to allow for an enhanced anti-tumor immune response following RT. Preclinical models strongly support this notion and clinical data is just emerging that suggests that this strategy may also be efficacious in the clinical setting.

One of the most successful regimens targeting intratumoral immunosuppression has been targeting immune suppression with checkpoint inhibitors which are agents that target the PD-1/PD-L1 and CTLA-4 pathways. Innate immune cells are one of the key sources of signal for the PD-1/PD-L1 pathway with dendritic cells and macrophage serving as one of the primary, non-tumor sources of PD-L1 in the tumor microenvironment. Thus, the underlying mechanism of checkpoint blockade likely involves disrupting the effects of innate immune cells on immune response in tumors. To date, an increasingly large amount of data has demonstrated the efficacy of using checkpoint inhibitors in the preclinical and clinical setting in combination with RT. As several excellent recent reviews have examined the role of combining checkpoint blockade with RT in detail, we will not discuss combinations with checkpoint blockade further here though it should be recognized that including one of these agents as a part of any immune-directed therapeutic regimen will be an important consideration for the foreseeable future (106, 107).

Beyond checkpoint blockade, macrophages serve as the main source of immunosuppression within the tumor microenvironment following RT. As evidence of the importance of macrophages, various studies have revealed a strong negative correlation between the presence of macrophages and survival in various solid tumors including breast, colon, bladder, and lung cancer (10–12, 108). As we described above, macrophages are often associated with resistance to radiotherapy and chemotherapy by providing both pro-survival signals and tissue repair functions that protect and/or repair the damage done by these therapies. Various studies have shown that macrophages, the most abundant cells of the tumor microenvironment, are altered by RT to support tumor growth after being damaged and sensing damage resulting from irradiation. For example, Leblond et al. found an increase in density of pro-tumor M2 macrophages in the tumor microenvironment post-RT in glioblastoma (109). Kioi et al. showed that the RT-recruited macrophages help rectify the damage done by RT by promoting vasculogenesis (14). Given, the pro-tumor role of macrophages following RT multiple groups have shown that blocking macrophage recruitment via targeting CD11b (16), CCL2 (81), or CSF-1R (4, 75, 100), enhance the efficacy of RT in preclinical murine models. For example, in a squamous cell carcinoma model Ahn et al. found that administration of a CD11b antibody enhanced the efficacy of RT by blocking myeloid cell recruitment to the tumor site after RT leading to delayed regrowth in part through impaired angiogenesis (16). Other studies have revelead that inhibition of macrophages following RT increases both the anti-tumor immune response (4) and prevents pro-tumor repair mechanisms such as angiogenesis and matrix remodeling (14, 16). Thus, these studies all demonstrate that targeting macrophages can synergize with RT, however, given the potentially positive role of macrophages in producing cytotoxic anti-tumor immune responses, other groups have sought to preserve the pro-inflammatory activation capacity of macrophages while preventing their suppressive differentiation to even further synergize with RT.

Given the successful preclinical models showing enhanced responses to RT in combination with agents that target macrophages including CSF-1R inhibitors and CD11b, several trials are currently underway to test the validity of this observation in human trials. Based on the work of Stafford et al. a trial using the small molecule inhibitor of CSF-1R (Pexidartinib, PLX3397, Plexxikon) in newly diagnosed glioblastoma in combination with standard chemotherapy and RT was opened and accruing (ClinicalTrials.gov, NCT01790503). Another group is also testing the CSF-1R inhibitor in combination with concurrent standard dose RT and androgen deprivation for localized unfavorable risk prostate cancer (ClinicalTrials.gov, NCT02472275). Interestingly, agents targeting tumor-associated macrophages such as the CCL2 inhibitor carlumab have had limited effect as single-agents (110) and in fact may only have efficacy when combined with other agents such as RT that perturb the tumor immune microenvironment (4, 111).

In order to preserve the macrophage capacity to activate anti-tumor immunity while preventing their differentiation into pro-tumor, immunosuppressive phenotypes, several groups including our own have examined the potential of targeting the pathways that lead to pro-tumor phenotypes in macrophages including IL-4 (4), arginase 1 (102), TGF-β (15), and Tyro3/Axl/Mer (TAM) tyrosine kinases (18, 101) in combination with RT. Targeting macrophage differentiation led to improved anti-tumor immunity, particularly cytotoxic CD8+ T cells, resulting in dramatically enhanced responses to RT. Though each of these strategies targets a distinct pathway found in myeloid-macrophages, they result in reduction but likely not elimination of immunosuppressive differentiation suggesting that even modest reductions in tumor-associated immunosuppression can have profound effects on therapeutic responsiveness to RT.

The findings from these preclinical studies targeting macrophage phenotype in combination with radiation are just beginning to be explored in the clinical trial setting. One promising target is TGF-β a cytokine for which several inhibitors have been developed. Though TGF-β has pleiotropic effects, its upregulation post-RT is one of the primary drivers of immunosuppression in the irradiated tumor microenvironment particular effects on the development of regulatory macrophages and T cells. Using an agent that binds all isoforms of TGF-β (fresolimumab, Sanofi-Aventis) in combination with SBRT for patients with metastatic breast cancer (ClinicalTrials.gov, NCT02538471), Formenti et al. found that the highest dose combination led to improved survival and systemic immune responses compared to lower doses (112). Other clinical trials testing TGF-β inhibition with RT and/or chemotherapy are currently underway in non-small cell lung cancer (ClinicalTrials.gov, NCT02581787), glioblastoma (ClinicalTrials.gov, NCT01220271), and hepatocellular carcinoma (ClinicalTrials.gov, NCT02906397). Other pathways targeting macrophage phenotypes have not yet been explored clinically, but the experience with TGF-β suggests that strategies that help create favorable macrophage phenotypes may mirror the preclinical data in improving the efficacy of RT.

## Conclusions

The innate immune system plays a critical role in regulating the response to RT from the recognition of RT-mediated tissue damage to shaping of the RT-mediated anti-tumor immune response. Strategies to augment the innate immune response have met with varying success clinically, however promising new strategies based on our improved understanding of innate immune biology such as STING agonists, adjuvants to enhance DC activity and anti-macrophage agents will undoubtedly shape future therapeutic approaches to combination therapies with RT.

## Author Contributions

TD and SS conceived this article. TD, RH, and SS wrote the article and designed the figures.

### Conflict of Interest Statement

The authors declare that the research was conducted in the absence of any commercial or financial relationships that could be construed as a potential conflict of interest.
